# Supplementary material for: BACH1 as a key driver in rheumatoid arthritis fibroblast-like synoviocytes identified through gene network analysis
Source: Life Sci Alliance. 2024 Oct 28;8(1):e202402808. doi: 10.26508/lsa.202402808 (PMC11519322; doi:10.26508/lsa.202402808)
Supplement: Supplementary file 1 [file LSA-2024-02808_TableS1.docx]

**Supplementary Materials**

# Supplementary Tables

**Table S1:** Top 30 differentially expressed genes in FLS RA *vs* OA, ranked by *t*-score . The *p*-values were not adjusted.

| |

Gene (top 15) *t*-score *p*-value LINC01600 4.70 6.22 × 10−5

SLC25A26 4.29 1.94 × 10−4

MMD -3.90 5.46 × 10−4

NUDT21 -3.77 7.74 × 10−4

ATP8A1 -3.76 7.94 × 10−4

GPR4 3.74 8.49 × 10−4

ARHGAP28 3.68 9.84 × 10−4

BIRC7 3.67 1.01 × 10−3

ALOX15B -3.63 1.13 × 10−3

STKLD1 -3.58 1.28 × 10−3

TMEM128 3.58 1.29 × 10−3

E2F1 3.55 1.38 × 10−3

PRKDC -3.53 1.47 × 10−3

PROS1 3.50 1.58 × 10−3

CNFN -3.47 1.69 × 10−3

Gene (rank 16-30) *t*-score *p*-value CPAMD8 -3.47 1.70 × 10−3

PRKG1 3.45 1.79 × 10−3

RAB15 -3.38 2.14 × 10−3

ACYP2 3.37 2.20 × 10−3

TRDN 3.37 2.23 × 10−3

SOAT2 3.37 2.23 × 10−3

NFATC3 3.36 2.29 × 10−3

E2F5 3.34 2.39 × 10−3

EGFL7 -3.33 2.47 × 10−3

MUSTN1 3.32 2.53 × 10−3

TRHDE 3.32 2.54 × 10−3

FAM47E 3.31 2.56 × 10−3

ANKRD11 -3.30 2.61 × 10−3

TMEM154 -3.30 2.62 × 10−3

SAMD4A -3.30 2.62 × 10−3
