# Supplementary material for: BACH1 as a key driver in rheumatoid arthritis fibroblast-like synoviocytes identified through gene network analysis
Source: Life Sci Alliance. 2024 Oct 28;8(1):e202402808. doi: 10.26508/lsa.202402808 (PMC11519322; doi:10.26508/lsa.202402808)
Supplement: Supplementary file 2 [file LSA-2024-02808_TableS2.docx]

**Table S2:** Top10 Pathway of FLS DEGs (1093 genes).

**Biological Process/Pathway Genes adj.** *p***-value**

Negative regulation of low-density lipopro- tein receptor activity.

Arrhythmogenic right ventricular cardiomy- opathy.

Ubiquinone and other terpenoid-quinone biosynthesis.

Axon guidance.

Hypertrophic cardiomyopathy.

Arginine and proline metabolism. Dilated cardiomyopathy.

Carnitine shuttle.

Regulation of vascular associated smooth muscle cell migration.

ECM-receptor interaction.

ABCA2, APP, ADIPOQ, PCSK9, ITGAV (5/7)

SLC8A3, DES, CDH2, SGCA, ITGA3, ITGA11, ITGB8, CACNA2D2, ITGAV, ITGA6, CACNA1D, ITGA5 (12/77)

NQO1, COQ2, TAT, COQ6 (4/11)

NTNG2, ROBO3, SEMA6D, SEMA3A, SEMA3B, NFATC3, PIK3CD, UNC5C, SEMA3E, SSH3, RHOD, RGMA, EFNB2, EFNA3, EFNB3, ABLIM3, RAC2, EPHB2, MET, PLXNA4 (20/182)

SLC8A3, PRKAA2, DES, SGCA, ITGA3, ITGA11, ITGB8, CACNA2D2, ITGAV, ITGA6, CACNA1D, ITGA5 (12/90)

GAMT, MAOB, CKM, SMOX, PYCR2, HOGA1, PRODH, L3HYPDH (8/50) SLC8A3, PLN, DES, SGCA, ITGA3, ITGA11, ITGB8, CACNA2D2, ITGAV, ITGA6, CACNA1D, ITGA5 (12/96)

PRKAA2, CPT2, THRSP, ACACB, ACACA (5/11)

FGF9, IGFBP5, ADIPOQ, DOCK7, PRKG1 (5/12)

LAMA5, SV2B, TNN, ITGA3, ITGA11, ITGB8, ITGAV, ITGA6, ITGA5, COL9A2, THBS1 (11/88)

0.035

0.231

0.231

0.231

0.265

0.299

0.299

0.317

0.345

0.353
