# Supplementary material for: BACH1 as a key driver in rheumatoid arthritis fibroblast-like synoviocytes identified through gene network analysis
Source: Life Sci Alliance. 2024 Oct 28;8(1):e202402808. doi: 10.26508/lsa.202402808 (PMC11519322; doi:10.26508/lsa.202402808)
Supplement: Supplementary file 3 [file LSA-2024-02808_TableS3.docx]

**Table S3:** List of the top TF regulators in FLS with a Z-statistics above 1, provided in parenthesis.

| **Rank 1-25** | **Rank 26-50** | **Rank 51-75** | **Rank 76-91** |
| --- | --- | --- | --- |
| RORC (3.39)  NKX2-1 (3.37)  HOXA1 (3.20)  ETV2 (2.67)  MITF (2.64)  SIX5 (2.61)  ELF5 (2.46)  ZBTB1 (2.42)  FOXC2 (2.27)  TBX4 (2.23)  CBFB (2.16)  BBX (2.16)  TAL1 (2.13)  ZBTB3 (2.11)  RARG (2.09)  ETV3 (2.04)  TLX3 (1.97)  HOXB3 (1.95)  PKNOX1 (1.95)  MECOM (1.94)  ELK4 (1.92)  HOXB7 (1.91)  DBX2 (1.91)  HLX (1.90)  TBX2 (1.90) | MNT (1.87)  BACH1 (1.83)  IKZF1 (1.82)  FEZF1 (1.82)  SOX10 (1.81)  PGR (1.76)  CREB3 (1.75)  FOS (1.72)  CEBPZ (1.66)  HOXB6 (1.66)  MEOX2 (1.64)  GABPA (1.64)  MEIS1 (1.63)  HOXB1 (1.63)  NR6A1 (1.62)  E4F1 (1.61)  ELF3 (1.60)  CDX1 (1.58)  ATF7 (1.58)  CLOCK (1.58)  ETV7 (1.57)  RFX5 (1.54)  HIF1A (1.52)  FOXI1 (1.50)  TBX5 (1.50) | ZBTB4 (1.49)  HNF1B (1.48)  IRX1 (1.47)  DPRX (1.45)  HLF (1.45)  IRF6 (1.43)  TFCP2 (1.43)  TGIF2LX (1.42)  HOXB8 (1.42)  GLIS1 (1.39)  ZNF713 (1.39)  NFE2 (1.38)  THRB (1.34)  ELF4 (1.34)  HLTF (1.32)  TGIF1 (1.29)  HOXB5 (1.26)  RUNX1 (1.27)  NPAS4 (1.26)  BARX2 (1.25)  HOXA2 (1.25)  E2F4 (1.25)  GCM2 (1.24)  ZKSCAN3 (1.21)  VTN (1.21) | ATF2 (1.20)  GSC2 (1.20)  NFYB (1.18)  IRF7 (1.18)  CREM (1.16)  MEIS2 (1.16)  NFAT5 (1.15)  SOX30 (1.15)  GATA2 (1.14)  NFATC1 (1.13)  FOSL1 (1.12)  CEBPB (1.11)  FOXO4 (1.09)  EN2 (1.09)  FLI1 (1.09) |
