# Supplementary material for: BACH1 as a key driver in rheumatoid arthritis fibroblast-like synoviocytes identified through gene network analysis
Source: Life Sci Alliance. 2024 Oct 28;8(1):e202402808. doi: 10.26508/lsa.202402808 (PMC11519322; doi:10.26508/lsa.202402808)
Supplement: Supplementary file 4 [file LSA-2024-02808_TableS4.docx]

**Table S4:** Top10 Pathway of FLS top TFs (185 TFs).

**Biological Process/Pathway**

Activation Of HOX Genes During Differen- tiation.

Regulation of myeloid cell differentiation. Anterior/posterior pattern specification.

Developmental Biology.

**Genes**

RARG, MEIS1, HOXA2, HOXB3, HOXA1, HOXB1, PKNOX1 (7/91)

NFE2, MEIS1, CBFB, HOXB8, MEIS2, RUNX1 (6/68)

CDX1, HOXB3, HOXB7, HOXB6, HOXB5 (5/63)

CEBPB, RARG, CBFB, HNF1B, GATA2, SOX10, FLI1, RUNX1, MEIS1, NR6A1, TAL1, HOXA2, HOXB3, HOXA1, HOXB1, PKNOX1 (16/1073)

RARG, TBX5, TBX4, TBX2 (4/36) ELF1, CBFB, RUNX1 (3/13)

FOSL1, ELK4, ATF2, CREB3, NFYB, NFATC1, FOS (7/219)

TBX5, RUNX1, TBX2 (3/21) ELF1, CBFB, RUNX1 (3/22)

MEIS1, HOXB8, MEIS2 (3/22)

**adj.** *p***-value**

0.000030

0.000037

0.000513

0.000764

Embryonic limb morphogenesis. Regulation of antigen receptor-mediated signaling pathway.

Human T-cell leukemia virus 1 infection.

Regulation of cardiac muscle cell prolifera- tion.

Regulation of response to cytokine stimu- lus.

Negative regulation of myeloid cell differ- entiation.

0.000942

0.001054

0.003470

0.003834

0.003834

0.003834
