# Supplementary material for: BACH1 as a key driver in rheumatoid arthritis fibroblast-like synoviocytes identified through gene network analysis
Source: Life Sci Alliance. 2024 Oct 28;8(1):e202402808. doi: 10.26508/lsa.202402808 (PMC11519322; doi:10.26508/lsa.202402808)
Supplement: Supplementary file 5 [file LSA-2024-02808_TableS5.docx]

**Table S5:** Top 100 TGs in FLS. The number in parenthesis corresponds to the number of key TF drivers (182 TFs with Z-statistics *>* 0.5) differentially targeting this gene (|*t*_diff-edge_|>1). Note that only genes differentially expressed between RA and OA FLS are considered here, |*t*_diff-expr_|>1.

| **Rank 1-25** | **Rank 26-50** | **Rank 51-75** | **Rank 76-100** |
| --- | --- | --- | --- |
| ALDH1A2 (67)  SYNE2 (64)  NF1 (62)  FOCAD (59)  EML1 (58)  ABCA5 (56)  DCDC1 (54)  TRPM8 (53)  COBLL1 (52)  HDAC6 (50)  VPS8 (50)  USP47 (49)  ASAP1 (49)  NOSTRIN (48)  TCF4 (48)  PTPN12 (47)  MSI2 (47)  CLTC (46)  DENND2B (46)  ADGRA3 (45)  SOX6 (45)  CBWD5 (44)  ANK3 (44)  SIPA1L1 (44)  TUT4 (44) | PAPOLA (44)  EFL1 (43)  UBR4 (43)  USP34 (43)  DLG2 (43)  ZNF532 (42)  TFDP2 (42)  CD163L1 (42)  TACC2 (42)  MPHOSPH9 (42)  GTDC1 (44)  EFCAB5 (44)  TM7SF3 (44)  ELAPOR2 (44)  ABI3BP (44)  FRMD3 (44)  GRAMD2B (40)  DNAH14 (40)  UTRN (40)  SKIL (40)  ROBO2 (40)  NFASC (39)  SEMA6A (39)  EHBP1 (39)  ATAD2B (39) | POT1 (39)  LPP (39)  ULK2 (38)  RNF220 (38)  DPYS (38)  SLC9B2 (38)  PAH (38)  EYA4 (37)  ITGAV (37)  SFPQ (37)  NUP205 (37)  ANKRD26 (67)  HERC4 (36)  RBM17 (36)  UVRAG (35)  TNIK (35)  MYBPC1 (35)  TAOK3 (35)  FN1 (34)  SLC11A2 (34)  STPG1 (34)  RPRD1A (34)  ATP6V0A1 (34)  GON4L (34)  TLN2 (34) | MET (34)  SORBS2 (34)  SHANK2 (33)  GALC (33)  RBMS1 (33)  RMDN1 (33)  ANGPT1 (33)  MAP4K5 (33)  CPNE4 (33)  CYLD (32)  CTNNA3 (32)  BCAS3 (32)  XPNPEP1 (32)  PHTF1 (32)  TRIM29 (32)  JKAMP (32)  TNIP3 (31)  MAN2A1 (31)  MAPK6 (31)  REXO2 (31)  SEMA6B (31)  NCAPD3 (31)  DDX46 (31)  TCERG1 (31)  CBWD6 (31) |
