# Supplementary material for: BACH1 as a key driver in rheumatoid arthritis fibroblast-like synoviocytes identified through gene network analysis
Source: Life Sci Alliance. 2024 Oct 28;8(1):e202402808. doi: 10.26508/lsa.202402808 (PMC11519322; doi:10.26508/lsa.202402808)
Supplement: Supplementary file 6 [file LSA-2024-02808_TableS6.docx]

**Table S6:** Top10 Pathway of FLS top TGs (211 genes).

**Biological Process/Pathway**

Positive regulation of GTPase activity.

**Genes adj.** *p***-value**

BCR, BCAS3, SIPA1L1, DOCK9, TBC1D5, 0.004845

NF1, PIP5K1A, ITGA6, SIPA1L2, MAPRE2, RGS6, MAP4K4 (12/214)

BCR, ACTN2, FN1, PIP5K1A, ITGA6 (5/34)

BCAS3, FGD5, SIPA1L1, DOCK9, RDX, NF1, ITGA6, MAPRE2, RGS6, MAP4K4 (10/189) TAOK3, DTNBP1, TNIK, MYO16, MAP4K4 (5/52)

ROBO2, SEMA6B, SEMA6A, PTPN2 (4/32) BCAS3, ANGPT1, NF1, PTPRM, PRCP, FOXP1 (6/89)

BCR, ACTN2, PIP5K1A (3/21) SUN3, NUP205, TPR, LEMD3 (4/48) MAPRE2, MAP4K4 (2/6)

ROBO2, PTPRM (2/6)

Cell-substrate junction assembly. Regulation of GTPase activity.

Plasma membrane bounded cell projection morphogenesis.

Negative regulation of chemotaxis. Regulation of endothelial cell migration.

Focal adhesion assembly. Nucleus organization.

Positive regulation of focal adhesion disas- sembly.

Retinal ganglion cell axon guidance.

0.017964

0.017964

0.086533

0.096032

0.096032

0.108547

0.108547

0.108547

0.108547
