# Supplementary material for: BACH1 as a key driver in rheumatoid arthritis fibroblast-like synoviocytes identified through gene network analysis
Source: Life Sci Alliance. 2024 Oct 28;8(1):e202402808. doi: 10.26508/lsa.202402808 (PMC11519322; doi:10.26508/lsa.202402808)
Supplement: Supplementary file 7 [file LSA-2024-02808_TableS7.docx]

**Table S7:** List of networks involved in our study. GIANT [[35](#_bookmark50)] RIMBANET and PPI were downloaded from public database while PANDA and LIONESS networks were computed specifically for this study.

| **Network(s) name** | **Type** | **Method** | **Used for** | **Number of networks** |
| --- | --- | --- | --- | --- |
| PANDA_FLS | Bipartite | PANDA, LIONESS* | TF reg. score | 18RA, 12OA |
| GIANT_DC | Bayesian | GIANT§ | KDA | 1 |
| GIANT_NKT | Bayesian | GIANT§ | KDA | 1 |
| GIANT_Monocyte | Bayesian | GIANT§ | KDA | 1 |
| GIANT_Tcell | Bayesian | GIANT§ | KDA | 1 |
| GIANT_Bcell | Bayesian | GIANT§ | KDA | 1 |
| GIANT_Fibroblast | Bayesian | GIANT§ | KDA | 1 |
| GIANT_Adipocyte | Bayesian | GIANT§ | KDA | 1 |
| GIANT_Tonsil | Bayesian | GIANT§ | KDA | 1 |
| GIANT_LN | Bayesian | GIANT§ | KDA | 1 |
| GIANT_Blood | Bayesian | GIANT§ | KDA | 1 |
| GIANT_Spleen | Bayesian | GIANT§ | KDA | 1 |
| RIMBANET_Multitissue | Bayesian | RIMBANET† | KDA | 1 |
| PPI | Undirected | StringDB† | KDA | 1 |

* Computed in this study from bulk RNAseq [[20](#_bookmark35)].

§ GIANT networks downloaded from <https://hb.flatironinstitute.org/download>.

† RIMBANET and PPI downloaded from <http://mergeomics.research.idre.ucla.edu/samplefiles.php>.
