# Supplementary material for: BACH1 as a key driver in rheumatoid arthritis fibroblast-like synoviocytes identified through gene network analysis
Source: Life Sci Alliance. 2024 Oct 28;8(1):e202402808. doi: 10.26508/lsa.202402808 (PMC11519322; doi:10.26508/lsa.202402808)
Supplement: Supplementary file 8 [file LSA-2024-02808_TableS8.docx]

**Table S8: list of KDA TF (174 TFs)** [[49](#_bookmark64)], ordered from highest to lowest score.

STAT1, IRF1, STAT3, NFKB1, NFKB2, RELB, RUNX3, IRF9, MAFB, JUNB, FLI1, BCL6, IRF8, ELF4, ETS2, STAT5A, STAT4, HHEX, ATF3, TFEC, HIF1A, CEBPD, MEF2C, EGR1, ETS1, IKZF1, FOSL2, RUNX1, TGIF1, FOS, EGR2, IRF7, LMO2, REL, STAT2, KLF4, STAT6, AHR, MITF, STAT5B, ETV6, ATF5, BATF, RBPJ, NR3C1, HLX, JUND, KLF2, BHLHE40, RELA, TCF4, XBP1, TCF7L2, IRF5, CREM, TFE3, JUN, FOXO1, SPI1, NR4A3, IRF2, MAFF, BACH1, RFX5, TBX21, TP53, CEBPB, NFATC1, CIC, GATA3, BACH2, GFI1, LEF1, MYC, MSC, IRF3, CEBPA, MAX, POU2F2, BATF3, NR4A2, CREB5, MTF1, ELK3, EPAS1, EGR3, FOSB, SP1, ENO1, NR4A1, ETV7, ELF1, HIVEP1, ERG, HIVEP2, HBP1, HOXB2, NR1H2, KLF5, MYBL1, ZNF274, PRRX1, DNMT1, VENTX, MEF2A, CBFB, NFE2, PBX3, SMAD3, KMT2A, ARNTL, TP63, ZNF281, KLF3, SREBF1, EOMES, CREB1, ZNF263, RORA, ATF4, ZNF32, ETV5, FOSL1, BCL11A, PLAGL1, TCF7, NR5A2, KLF13, TBX19, USF2, MEOX1, E2F4, NFAT5, SOX4, NFE2L1, TWIST1, CEBPE, PPARD, DDIT3, PATZ1, FEV, FOXB1, FOXC2, FOXF1, NR2F2, NR2C2, ATF6, FOXO4, TLX1, CREB3, ARID3A, ARID5B, TGIF2, FOXJ2, ATF2, TFEB, IRF6, RARA, HSF4, ERF, NFIB, RXRA, ZBTB7A, PRDM1, AIRE, PROP1, SOX9, MSX1, PITX3, RUNX2, HES1, ZNF35, SOX18, IRF4.
