# Supplementary material for: BACH1 as a key driver in rheumatoid arthritis fibroblast-like synoviocytes identified through gene network analysis
Source: Life Sci Alliance. 2024 Oct 28;8(1):e202402808. doi: 10.26508/lsa.202402808 (PMC11519322; doi:10.26508/lsa.202402808)
Supplement: Supplementary file 9 [file LSA-2024-02808_TableS9.docx]

**Table S9:** The 28 key TFs implicated in the regulation of RA FLS, as identified in our analyses, are ranked by Z-statistics in each co-regulation cluster. The degree in the TF co-regulation network Figure [3](#_bookmark4)A is also provided.

| TF | Cluster | FLS score  (dGRN Z-statistics) | Degree |
| --- | --- | --- | --- |
| BACH1 |  | 1.83 | 15 |
| FOS |  | 1.72 | 10 |
| HIF1A |  | 1.52 | 15 |
| TGIF1 |  | 1.39 | 17 |
| CREM |  | 1.16 | 13 |
| FOSL1 |  | 1.12 | 14 |
| FLI1 |  | 1.09 | 15 |
| BHLHE40 STAT1 EGR2 | 1 | 0.98  0.87  0.83 | 19  18  17 |
| NR4A1 |  | 0.75 | 17 |
| FOSL2 |  | 0.71 | 13 |
| JUNB |  | 0.68 | 14 |
| HIVEP1 |  | 0.65 | 16 |
| PLAGL1 |  | 0.62 | 21 |
| ENO1 |  | 0.59 | 15 |
| HLX |  | 1.90 | 12 |
| NFATC1 IRF7 | 2 | 1.23  1.18 | 13  12 |
| HOXB2 |  | 0.54 | 3 |
| CBFB | 3 | 2.16 | 7 |
| RUNX1 |  | 1.27 | 8 |
| RFX5 |  | 1.54 | 2 |
| ETV7 | 4 | 1.57 | 10 |
| ELF1 |  | 1.03 | 8 |
| ELF4 |  | 1.34 | 4 |
| MITF | 5 | 2.64 | 10 |
| IKZF1 |  | 1.93 | 5 |
