# Supplementary material for: BACH1 as a key driver in rheumatoid arthritis fibroblast-like synoviocytes identified through gene network analysis
Source: Life Sci Alliance. 2024 Oct 28;8(1):e202402808. doi: 10.26508/lsa.202402808 (PMC11519322; doi:10.26508/lsa.202402808)
Supplement: Supplementary file 10 [file LSA-2024-02808_TableS10.docx]

**Table S10: List of BACH1 gene targets in the FLS network (131 genes)**, ranked by most to least differentially targeted in RA *vs* OA. Only genes with (|*t*_diff-edge_|>1 and |*t*_diff-expr_|>1) are shown in the list.

SLC25A44, TANGO2, ALDH1A2, LINC01630, PPIP5K1, AARS2, SLC9A3R2, CD163L1, AMPH, ZFAND2B, GOLGA7, EFCAB7, OGDHL, NANOS3, XPNPEP1, SYTL2, PIGA, GRM4, KITLG, NBEAL2, CLTRN, ZSCAN31, PHEX, TNIP1, PXDNL, TUBBP5, ELAPOR2, GPR160, ATAD2B, RSPH14, TUT7, NFASC, CYB5A, KLHL3, DECR2, ACSL6, RNF19A, KLRG1, PRIM1, UBE2I, NEB, FEZ1, CTPS2, GALNT3, BUD31, HADHB, CDK7, TM4SF1, CEP350, PDZRN3, TMEM244, LPP, ACOT11, UBR4, USP47, CHCHD6, SP5, EXOC7, ENPP2, TAF1, CFH, S100B, ICAM2, GGA1, MAPK6, FGF1, SESN1, CYP4F3, CCDC150, TMEM106C, LINC00313, CBFB, TMTC1, CPT1C, SLC4A3, ZFPM2, FBRSL1, CCDC190, TREML3P, DGCR6L, TMEM161A, NFE2L2, FSTL3, RBM4B, AKAP7, MYO16, GLB1L2, RIPOR3, DDX20, ATP10D, MYO9B, ATOX1, PI4KB, ROR2, CYB5R2, SSC5D, TMEM63B, LMBRD1, AP4B1, DRG1, SAYSD1, ABCC3, ATP13A4, HTATIP2, SH3TC1, CLEC3A, PDZD2, RRM1, RDX, SLC7A11, OXCT1, CD109, NTNG1, USP34, CPED1, MYOCD, ADAMTS3, CHRNA6, MED8, ALDH3A2, ZBTB9, IGSF10, FBXL5, ABCC8, DBF4B, MVD, NFIC, UBE2D3, GRHPR, NAXE, GCLC.
