# Supplementary material for: BACH1 as a key driver in rheumatoid arthritis fibroblast-like synoviocytes identified through gene network analysis
Source: Life Sci Alliance. 2024 Oct 28;8(1):e202402808. doi: 10.26508/lsa.202402808 (PMC11519322; doi:10.26508/lsa.202402808)
Supplement: Supplementary file 11 [file LSA-2024-02808_TableS11.docx]

**Table S11:** Significant pathways of BACH1 targets (131 genes).

**Biological Process/Pathway**

Fatty acid degradation. Ferroptosis.

Valine, leucine and isoleucine degradation. Metabolism of lipids.

**Genes**

ALDH3A2, HADHB, ACSL6, CPT1C (4/43) GCLC, ACSL6, SLC7A11 (3/41) ALDH3A2, HADHB, OXCT1 (3/48)

ABCC3, UBE2I, CD163L1, CYP4F3, ACOT11, ACSL6, MED8, ALDH3A2, HADHB, OXCT1, MVD, PI4KB, DECR2 (13/732)

ALDH3A2, HADHB, CYP4F3, ACOT11, ACSL6, DECR2 (6/173)

NAXE, CD163L1, PPIP5K1, OXCT1, ENPP2, DECR2, SLC25A44, ABCC3, CYB5A, RRM1, UBE2I, ABCC8, NANOS3, CYP4F3, ACOT11, ACSL6, MED8, CTPS2, ALDH3A2, HADHB, GRHPR, GCLC, LMBRD1, MVD, PI4KB (25/2049)

**adj.** *p***-value**

0.018154

0.124481

0.130240

0.261402

Fatty acid metabolism.

Metabolism.

0.261402

0.269435
