# Supplementary material for: BACH1 as a key driver in rheumatoid arthritis fibroblast-like synoviocytes identified through gene network analysis
Source: Life Sci Alliance. 2024 Oct 28;8(1):e202402808. doi: 10.26508/lsa.202402808 (PMC11519322; doi:10.26508/lsa.202402808)
Supplement: Supplementary file 12 [file LSA-2024-02808_TableS12.docx]

**Table S12:** Differentially expressed genes between siBACH1 and siCTL (adjusted *p*-value *>* 0.05).

Gene (*downregulated*) log2fold *p*-value CBFB -1.55 2.92 × 10−12

SLC35A5 -2.71 2.44 × 10−11

BACH1 -2.53 2.23 × 10−9

NUDT15 -2.13 5.50 × 10−9

EHD3 -1.33 1.65 × 10−8

PLA2G12A -1.74 4.46 × 10−8

BICD2 -1.07 1.47 × 10−7

N6AMT1 -1.55 9.09 × 10−7

STRADB -1.90 9.16 × 10−7

SYNM -1.04 2.85 × 10−6

FBXL17 -1.25 3.54 × 10−6

SLC2A3 -1.00 3.96 × 10−6

C11orf95 -1.05 6.61 × 10−6

PRKAG2 -1.08 7.00 × 10−6

DICER1 -1.48 7.65 × 10−6

SENP1 -1.62 1.56 × 10−5

TNC -0.99 2.14 × 10−5

TOR1B -1.03 2.89 × 10−5

ARHGEF3 -1.14 3.91 × 10−5

RSPO2 -1.02 4.70 × 10−5

FAM134B -1.10 9.65 × 10−5

GOLT1B -2.01 9.87 × 10−5

MIB1 -1.52 1.24 × 10−4

DBP -1.18 1.32 × 10−4

Gene (*upregulated*) log2fold *p*-value HMOX1 2.00 1.09 × 10−16

RP11-863P13.4 2.72 5.19 × 10−15

RP11-863P13.3 1.76 3.32 × 10−10

TRIB3 1.38 8.19 × 10−8

ZNF469 1.40 1.57 × 10−7

MIR22HG 1.15 2.28 × 10−6

SLC7A5 1.15 2.92 × 10−6

RP11-863P13.5 2.45 4.40 × 10−6

TSPAN2 2.14 7.69 × 10−6

MEG9 1.63 5.07 × 10−5

SLC6A9 0.99 5.37 × 10−5

CHAC1 1.09 6.35 × 10−5

KRTAP1-5 1.16 7.07 × 10−5

ADM2 1.06 7.46 × 10−5
