# Supplementary material for: BACH1 as a key driver in rheumatoid arthritis fibroblast-like synoviocytes identified through gene network analysis
Source: Life Sci Alliance. 2024 Oct 28;8(1):e202402808. doi: 10.26508/lsa.202402808 (PMC11519322; doi:10.26508/lsa.202402808)
Supplement: Supplementary file 13 [file LSA-2024-02808_TableS13.docx]

**Table S13:** Top10 Pathway analysis of siBACH1 silencing (740 genes).

**Biological Process/Pathway Genes adj.** *p***-value**

Intrinsic apoptotic signaling pathway in re- sponse to endoplasmic reticulum stress.

Intrinsic apoptotic signaling pathway.

Response of EIF2AK1.

Response to endoplasmic reticulum stress.

Extracellular structure organization.

Amino acid import across plasma mem- brane.

Response to unfolded protein.

L-aspartate transmembrane transport. Positive regulation of myoblast prolifera- tion.

PERK-mediated unfolded protein response.

PPP1R15A, DDIT3, ITPR1, TNFRSF10B, BAX, TRIB3, BAK1, CHAC1, BBC3, ATF4 (10/29)

PPP1R15A, ITPR1, IFI6, TNFRSF10B, TN- FRSF1B, BBC3, HIPK2, DDIT3, BAX, TRIB3, CHAC1, BAK1, SCN2A, FNIP2, ATF4, EPHA2 (16/102)

PPP1R15A, DDIT3, TRIB3, CHAC1, ATF5, ATF4 (6/15)

PPP1R15A, JUN, ITPR1, TNFRSF10B, THBS1, BBC3, HERPUD1, TMX1, DDIT3, BAX, TRIB3, CHAC1, BAK1, RNF185, ATF4 (15/110)

FBN2, COL15A1, ECM2, LAMC3, MMP1, ITGB3, TNC, F11R, THBS1, TGFBR1, COL1A1, ADAMTS15, COL3A1, SH3PXD2A, MMP16, COL5A3, SPP1, MMP19, COL9A3, ITGB6 (20/216)

SLC7A5, SLC6A9, SLC1A3, SLC1A4, SLC3A2, SLC1A5 (6/24)

HSPA8, HSPB8, DDIT3, HSPA4L, BAX, BAK1, TOR1B, HERPUD1 (8/49)

SLC1A3, SLC1A4, SLC1A5 (3/5) ATF2, SOX15, GATA6 (3/5)

IGFBP1, DDIT3, ATF4, HERPUD1 (4/11)

0.000134

0.001431

0.009375

0.012098

0.089010

0.089010

0.139243

0.139243

0.139243

0.139243
