# Supplementary material for: BACH1 as a key driver in rheumatoid arthritis fibroblast-like synoviocytes identified through gene network analysis
Source: Life Sci Alliance. 2024 Oct 28;8(1):e202402808. doi: 10.26508/lsa.202402808 (PMC11519322; doi:10.26508/lsa.202402808)
Supplement: Supplementary file 14 [file LSA-2024-02808_TableS14.docx]

**Table S14:** The two independent list of RA associated genes used in the Key Driver Analysis (KDA) [[49](#_bookmark64)].

### DEG_list (93 genes):

RFX5, IRF9, TFEC, DDX60, TNFAIP6, TNFSF10, DRAM1, NMI, TNFAIP8, PLEKHO1, GZMA, IL2RG, SAMSN1, SEL1L3, LEPROTL11, ECE1, TRPV2, ENTPD1, JAK2, SLAMF8, CCL18, LY96, GZMB, TRAF3IP3, AKAP1, LPXN, PTTG1, SYK, LRMP, ANK3, CTSH, S100A8, SDC1, CECR1, AIM2, TAPBPL, GPR65, PRDX6, NKG7, COMMD8, OPN3, NAT1, NAGA, OSBPL3, CD38, IL32, DDX24, TLR7, EVI2A, GUCY1B3, MGAT4A, SEMA4D, CD8A, GALNT6, LPIN3, SLC38A6, PLCG2, QPCT, TXNDC9, IFIT1, RFTN1, TRIM21, TNS3, SRRM2, AREL1, KIAA0125, LRRC15, LCK, DNAJC15, SIRPG, FAS, GZMK, GZMH, SYNGR2, ADAMDEC1, C1GALT1C1, UCP2, FKBP11, IL21R, CXCL9, DENND1B, RASGRP1, PSMB8, CSF1R, SMCO4, TPD52, HLA-DOB, PRKCB, ALOX5, CLIC1, MYC, IRF8, PTPRC, GSN.

### Litt_list (259 genes):

VEGFA, SAA1, CLEC16A, IL1RN, CXCR4, IL19, IL17A, IL4, IL18, CAT, FCGRT, CCL2, HSPD1, LTA, IFNG, MECP2, CXCL10, CD14, CCL5, PIP4K2C, ALB, JAK3, TLR4, IL6, IL10, TP53, TGFB1, SPP1, IL1A, IL1B, CD19, GPX3, CYP3A4, GGT6, CCR5, NFKBIA, CCR7, CXCR3, HMOX1, IL6ST, CASP8, TAP1, CD80, IGF1, RBPJ, CCL20, PADI1, ICAM1, FAS, FLNB, PADI6, CXCL16, AGBL2, COMP, PADI3, IL15, DHODH, IL13, MMP9, SAG, VIM, CXCR5, TNFRSF1B, EOMES, LPP, MTF1, PRDM1, ILF3, HLA-DRB1, CDK2, IQGAP1, C2, C5, TRAF1, MBL2, BCL2L15, UBASH3A, PXT1, IKZF3, CSF2, IRF8, ARAP1, MSH5, CTLA4, CCR6, C5orf30, IL11RA, TRAF6, LBH, ETS1, RASGRP1, NFKBIL1, ANKRD55, AIRE, KIAA1109, LST1, SMG7, VTCN1, CD40, ABCB1, GSDMB, HLA-DQA1, IFNGR2, CARD8, CXCL13, NOTCH4, PRRC2A, PLD4, FADS2, PTPN11, IRF5, LY6G5C, RCAN1, HLA-DPB1, CFB, HLA-DMB, LOC100506023, CCL19, FOXO1, PRMT1, IL2RB, BACH2, IRF4, CD2, CCL27, PSMB9, PTPN2, NFKBIE, CCL21, PADI4, CLIC1, LOC145837, PRKCQ, BLK, BCL3, DCLRE1C, HLA-DMA, RUNX1, HLA-DPA1, PUS10, FAM107A, BAD, SWAP70, RCOR1, NGF, BAG6, CLNK, APOM, RAD51B, TNFRSF14, UBE2L3, TAGAP, PSMB8, COG6, BAX, ZNF438, TNPO3, CALCR, ATM, GATSL3, AFF3, RELA, CD28, DDX6, IL21, IL20RA, HSPA14, FADS1, RTKN2, TNFAIP3, CD5, TXNDC11, ETV7, PAPOLG, FGFR1OP, FCGR2B, ICOS, STAT4, P2RY10, PANK4, FCGR2A, TEC, REL, HLA-C, ATG5, CDK5RAP2, CFLAR, C1QBP, PDE2A, PRKCB, PTPRC, CD83, PLCL2, ISG20, MICA, SPRED2, TYK2, TAPBP, CEP57, FADS3, ANXA3, FNDC1, IL2RA, PTPN22, IL6R, CD226, TAP2, JAZF1, TPD52, HLA-DOB, IL23R, KIF5A, ARID5B, MYC, CSF3, GATA3, TNF, SYNGR1, ANXA6, FCRL3, DPP4, AHNAK2, CSNK2B, ICOSLG, DOK6, CELF2, AIF1, LY6G6F, ACOXL, NCF2, PADI2, WDFY4, GPANK1, YDJC, MMEL1, CDK6, PPIL4, PXK, IL2, LY6G6C, SLAMF6, CD84, ZMIZ1, PRKCH, FAM167A, SH2B3, HLA-B, IRAK1, HLA-DQB1, HLA-A.
